# A multi-matrix workflow for isolation, characterization, and biobanking of antimicrobial- resistant bacteria from One Health surveillance samples

**DOI:** 10.3389/fmicb.2026.1855350

**Published:** 2026-09-23

**Authors:** Alexandra Bahr, Stefan E. Heiden, Dennis Karnatz, Susanne Sievers, Claudia Hirschfeld, Lorenzo Lagostina, Pacôme Monemo, Fabian H. Leendertz, Fee Zimmermann, Katharina Schaufler

**Affiliations:** 1 Department of Epidemiology and Ecology of Antimicrobial Resistance, Helmholtz Centre for Infection Research (HZI), Helmholtz Institute for One Health (HIOH), Greifswald, Germany; 2 Department of Microbial Physiology and Molecular Biology, Institute of Microbiology, University of Greifswald, Greifswald, Germany; 3 Department of Ecology and Emergence of Zoonotic Diseases, Helmholtz Centre for Infection Research (HZI), Helmholtz Institute for One Health (HIOH), Greifswald, Germany; 4 Centre Hospitalier et Universitaire de Bouaké (CHU de Bouaké), Bouaké, Côte d’Ivoire; 5 Université Alassane Ouattara de Bouaké, Bouaké, Côte d’Ivoire; 6 One Health Surveillance Research Group, Helmholtz Centre for Infection Research (HZI), Helmholtz Institute for One Health (HIOH), Greifswald, Germany; 7 University Medicine Greifswald, Greifswald, Germany

**Keywords:** antimicrobial resistance (AMR), AMR bacteria, bacterial isolation, culture-based workflows, One Health, surveillance

## Abstract

**Objectives:**

Standardized protocols for the isolation of antimicrobial-resistant (AMR) bacteria are typically optimized for individual sample types, limiting comparability across One Health surveillance domains. Here, we describe a multi-matrix workflow for the isolation, characterization, and biobanking of AMR bacteria (*Enterobacterales*, *Pseudomonadales* and *Staphylococcus aureus*) from diverse One Health sample types, with the aim of enabling reproducible and comparable AMR surveillance across sectors and settings.

**Objective:**

The workflow integrates common methodological principles, including enrichment media, defined combinations of selective and chromogenic agar plates, phenotype-based colony selection, antimicrobial susceptibility testing, and whole-genome sequencing–ready isolate preparation. A structured picking scheme enables the systematic selection of multiple isolates per phenotype, allowing resolution of within-sample and within-species diversity. The protocol is applicable to swabs, fecal material, water, soil, insects, and food samples and is designed to be scalable and adaptable to laboratories with varying levels of infrastructure.

**Results, application and demonstration:**

Application of the workflow to samples of varying complexity demonstrates its capacity to recover multiple AMR bacterial species and distinct strains across matrices. More complex samples yielded a higher number of isolates, and the picker scheme enabled discrimination of phenotypically and genotypically distinct strains within individual samples, illustrating the protocol’s resolution and robustness.

**Conclusion:**

This multi-matrix workflow provides a practical and reproducible approach for the isolation and biobanking of AMR bacteria across heterogeneous One Health samples. Its simplicity, adaptability, and cross-matrix comparability support its use in both high-throughput laboratories and resource-limited settings, thereby facilitating integrated AMR surveillance and comparative analyses at broader scales.

## 1 Introduction

Antimicrobial resistance (AMR) is widely recognized as one of the foremost threats to global public health, undermining the effectiveness of life-saving antimicrobial therapies. Recent global estimates suggest that as many as 4.95 million deaths in 2019 were associated with drug-resistant infections, of which 1.27 million were directly attributable to resistance itself, highlighting the burden of AMR on healthcare systems worldwide (Murray, 2022). Robust, actionable data are essential to inform effective interventions and policy responses to this crisis. The spread of AMR across clinical, agricultural, and environmental domains underscores the critical need for comprehensive and sustained surveillance systems. Surveillance enables the systematic collection, analysis, and interpretation of AMR and antimicrobial use data, providing evidence for national and global strategies aimed at mitigating resistance, guiding infection prevention and control efforts, and evaluating the impact of interventions over time. The World Health Organization’s Global Antimicrobial Resistance and Use Surveillance System (GLASS) exemplifies the international commitment to building harmonized surveillance frameworks, promoting standardized methodologies for data collection and analysis across diverse settings (WHO, 2017, 2021). A key component of such efforts is the laboratory workflow used to detect, isolate, and characterize AMR bacteria (ARB). Standardization of surveillance protocols is essential to ensure that data derived from different specimen types, laboratories, and geographical regions can be meaningfully compared. Without consistent sampling strategies, laboratory workflows, and susceptibility testing criteria, variation in methodological approaches can introduce artefactual differences that obscure true epidemiological patterns (Rempel and Laupland, 2009; Weller et al., 2024). To generate reliable, comparable data, these workflows must be standardized, scalable, and adaptable to different sample types and settings, while remaining compatible with downstream analyses such as whole-genome sequencing (WGS) and metagenomics. However, most published protocols focus on a single matrix—such as clinical swabs, water, or soil—and have been optimized for matrix-specific challenges and organisms of interest. Armalytė et al. (2019) describes the isolation of ARB from soil samples. Tryptic soy agar with a selection pressure is used for this purpose. Dominant colonies are selected for further isolation; however, on this agar, it is not possible to distinguish between the individual species based on morphology (Armalytė et al., 2019). Mahmud et al. (2023) also use solid culture media for isolation, but without selection pressure. In this study, resistance screening is performed at a later stage, allowing for the isolation of non-resistant bacteria as well. Both studies use different media for bacterial culture (Mahmud et al., 2023). Recent work on surveillance network standardization has emphasized the importance of harmonized data management, quality control, and inter-laboratory comparability to improve the reliability of AMR estimates and support coordinated global responses. Critically, the ability to compare AMR data across surveillance initiatives depends on ensuring that observed differences in isolated bacteria and detected genotypic AMR reflect true biological variation rather than methodological discrepancies. This necessitates rigorous adherence to standardized protocols for specimen handling and bacterial isolation, alongside quality assurance measures that mitigate bias and variability between laboratories. To address this gap, we developed a harmonized, multi-matrix workflow for the isolation, phenotypic characterization, and long-term storage of antimicrobial-resistant bacteria and original samples from swabs, fecal matter, water, insects, and soil. While established protocols are available for the isolation of ARB from individual sample matrices, these workflows are typically optimized for specific sample types and therefore limit methodological comparability across One Health studies. Our protocol integrates these matrix-specific approaches into a single workflow, enabling the consistent processing of diverse human, animal, food, and environmental samples while maintaining comparable cultivation and downstream characterization strategies. This protocol has been refined and validated at the Helmholtz Institute for One Health (HIOH) through multiple AMR surveillance projects conducted in diverse environmental and epidemiological settings, resulting in the biobanking of thousands of original samples and bacterial isolates from different geographic regions. Central to the workflow is the systematic capture of phenotypic diversity through selective cultivation, colony morphology-based picking, and the preparation of high-quality, WGS-ready isolates (Figure 1). In contrast to conventional matrix-specific workflows, the colony-picking strategy enables the targeted selection of bacterial isolates according to predefined resistance phenotypes before downstream characterization, facilitating the generation of comparable isolate collections across different sample types. To our knowledge, this is the first comprehensive protocol describing a harmonized workflow for the isolation, characterization, and biobanking of antibiotic-resistant bacteria belonging to the orders *Enterobacterales* and *Pseudomonadales*, as well as Gram-positive staphylococci (primarily MRSA), across such a broad range of One Health sample types, thereby providing a reproducible framework for integrated AMR surveillance at a broader scale.

**FIGURE 1:**
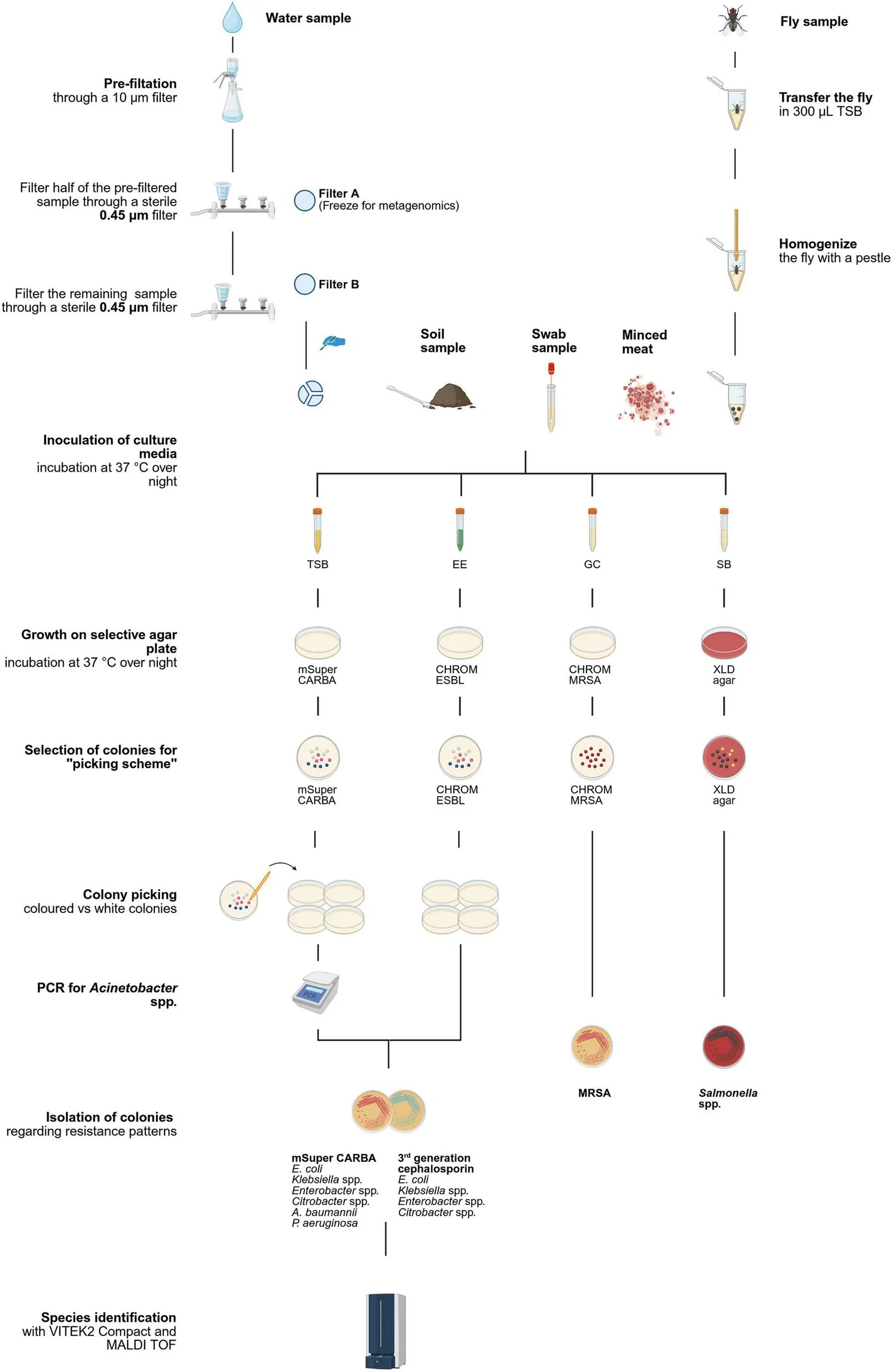
Schematic overview of the protocol from sample processing to species identification. TSB: tryptic soy broth; EE: *Enterobacteriaceae* enrichment broth; GC: Giolitti–Cantoni broth; SB: Selenite broth; ESBL: extended-spectrum beta-lactamase. Created in BioRender. Bahr, A. (2026) https://BioRender.com/tknl374.

## 2 Materials and equipment

This workflow requires the following reagents, consumables, and equipment. All microbiological work should be carried out using appropriate personal protective equipment and in accordance with biosafety level 2 practices.

### 2.1 Sample transport media

- eSwab (MAST Diagnostica GmbH, United Kingdom, 80490CEA)
- eNAT (MAST Diagnostica GmbH, United Kingdom, 80608CSR)
- Semi-solid Amies agar swab (e.g., Sarstedt, Germany, 80.1362)
- 50 mL tube (e.g., Falcon tube)
- Sterile bottle [glass or polyphenylenether (PPE)]

### 2.2 Reagents and media

- Tryptic soy broth (TSB, e.g., from Carl Roth, Germany)
- Granucult^®^ MOSSEL *Enterobacteriaceae* enrichment broth (EE, Merck-Millipore, Germany, 69965-500G)
- Selenite broth (SB, e.g., from Thermo Fisher Scientific, USA)
- Tryptic soy agar (TSA, e.g., from Carl Roth, Germany)
- Xylose lysine desoxycholate (XLD) agar (e.g., from Carl Roth, Germany)
- CHROMagar™ ESBL (MAST Diagnostica GmbH, United Kingdom, 15ESRT3)
- CHROMagar™ mSuperCARBA (MAST Diagnostica GmbH, United Kingdom, 15SC173)
- CHROMagar™ MRSA (MAST Diagnostica GmbH, United Kingdom, 15MR502)
- CHROMagar™ Acinetobacter base (MAST Diagnostica GmbH, United Kingdom, 15AC092)
- CHROMagar™ Serratia (MAST Diagnostica GmbH, United Kingdom, 15SM302)
- Tellurite (e.g., from OXOID, USA)
- Glycerol (e.g., from Carl Roth, Germany)
- Sodium biselenite (e.g., from OXOID, USA)
- Ciprofloxacin hydrochloride (e.g., from Serva, Germany)
- Milli-Q water for preparing media and solutions

### 2.3 Consumables

Unless otherwise specified, all consumables can be purchased from Sarstedt (Germany), for example.

- Sterile 1.5- and 2-mL tubes
- Barcoded cryotubes (e.g., SAFE^®^ LX1000, SAFE^®^ XLX500, LVL-technologies, Germany)
- Cryotubes with 200 μL sterile glycerol
- Sterile cultivation tubes
- Sterile serological pipettes
- Sterile inoculation loops (10 μL)
- Sterile toothpicks
- Sterile forceps
- Sterile scalpel
- Sterile scissors
- Sterile spatulas
- Sterile pestle
- Petri dishes (90 mm diameter)
- Filter (10 and 0.45 μm pore-size, Merck-Millipore, Germany)
- Sterile cotton swabs

### 2.4 Equipment

- Class II biosafety cabinet (BSL-2 compliant)
- AxonLab densitometer or equivalent turbidity measurement device
- Benchtop centrifuge (≥13,000 rpm)
- Incubator (37 °C, 42 °C)
−70 °C or −80 °C ultra-low temperature freezer
- VITEK2 Compact (bioMérieux, France) or equivalent for automated species identification and antimicrobial susceptibility testing (AST)
- MALDI-TOF MS (optional, for additional isolate identification)
- Analytical Profile Index (API-Test) and agar diffusion test (if no automated system is available)
- Vortexer
- Pipettes and sterile filter tips
- Water filtration unit (e.g., from Merck-Millipore, Germany)
- Vacuum pump (e.g., from Sartorius, Germany)
- Bunsen burner
- Anaerobic jars (e.g., from Sigma-Aldrich, USA)
- Fly trap and food bait (e.g., cat food)

## 3 Methods

### 3.1 Sampling

The sampling of One Health samples varies depending on the type of sample. Different types of One Health samples were taken in this study (Table 1). Here, swab samples were taken as smears; in animals (domestic and wild animals), these were nasal and rectal/cloacal swabs. We used eSwabs for the cultivation and isolation of bacteria from the swab, and also an eNAT swab for molecular biological analyses such as metagenomics. Commonly, fecal samples, both from humans and animals, were collected with a sterile spatula and placed into sterile containers (e.g., Falcon tube or cup). For this study, fecal samples from wild birds were collected using swabs. To do this, the swab was moistened with Amies medium and then rubbed briefly over the bird feces. In general, a sterile 1-liter bottle and gloves are required to collect water samples. Surface water samples were collected from the upper layer (20 cm) of the respective water bodies. Soil samples were obtained using a sterile spatula or an appropriate soil corer. For loose soil, approximately half of a 50 mL Falcon tube was filled using a sterile spatula. Flies were collected using a baited net trap that allowed entry but prevented escape. The trap was deployed at the sampling site for 20–30 min. Captured flies were subsequently transferred into sterile Falcon tubes. All samples were transported to the laboratory under refrigerated conditions and stored at 4 °C until further processing.

**TABLE 1:** Overview of different sample types used in this study.

Sample group	Sample type	Number of samples	Sample origin	Country
Environment	Soil	1	Farmland	Germany
Animal	Meat	1	Supermarket	Germany
Animal	Fecal swab	2	Wild bird	Germany
Animal	Flies	2	Taï national park	Côte d’Ivoire
Environment	Surface swab	1	Hospital	Côte d’Ivoire
Environment	Water	2	Hospital	Côte d’Ivoire

### 3.2 Storage of the different samples

The samples can be stored for varying lengths of time before they are processed. Water samples should ideally be processed immediately upon arrival at the laboratory, but no later than 24 h after arrival. This is because the microbial community composition of water samples can change rapidly once removed from their natural environment, due to alterations in oxygen availability and nutrient conditions. Swab samples, on the other hand, are usually stored in a suitable preservation medium in which the bacteria can survive for a longer period. For research purposes, swab samples may be stored for defined periods to assess bacterial recoverability. Ideally, swab samples should be processed within 2 months of sampling. Soil, fly, and fecal samples should be analyzed within a week to minimize changes to the microbiome. Retention periods are based on empirical data and the complexity of the samples. Samples with higher bacterial diversity and those stored without preservatives should be processed soon after sampling.

### 3.3 Biobanking

For long-term storage, the samples can be placed in a Hamilton Biobank system (SAM HD Pro, Hamilton Bonaduz AG, Switzerland) or similar storage systems for compact and easy storage and documentation, but a −70 °C or −80 °C freezer may be used as an alternative. Where feasible, both original material and cultivated isolates are archived. For example, for original soil and fecal samples, a sterile spatula is used to transfer some of the material into barcode-labeled tubes (LX/SX#). These are then stored at −70 °C or −80 °C. For fly, swab, and water samples, bacterial isolation requires the entire original sample; therefore, no aliquot of the raw material can be archived. For water samples, biomass enriched on a 0.45 μm membrane filter is transferred to the biobank. In addition, biobank samples are generated from cultivable microorganisms for all sample types. To this end, samples are incubated in TSB medium following the preparation steps described for Day 1 until a turbidity of 4.5–6.0 McFarland is reached. Subsequently, 800 μL of the culture is aseptically transferred into sterile, barcode-labeled LX1000 tubes containing 200 μL glycerol. The suspension is mixed thoroughly by repeated pipetting and immediately stored at −70 °C or −80 °C.

### 3.4 Bacterial isolation

This protocol is designed to isolate, characterize, and biobank antibiotic-resistant bacteria (ARB) belonging to the order *Enterobacterales* and *Pseudomonadales*, as well as Gram-positive staphylococci (primarily MRSA), from diverse One Health sample matrices. Tryptic soy broth (TSB), a nutrient-rich medium, supports the cultivation of *Pseudomonadales* and other heterotrophic bacteria. *Enterobacteriaceae* enrichment broth (EE) is used for the selective enrichment of *Enterobacteriaceae*, while selenite broth (SB) is applied to enrich *Salmonella* spp. Giolitti–Cantoni broth (GC) is used for the enrichment of Gram-positive bacteria. Owing to the heterogeneity of the sample matrices, initial processing steps differ depending on the sample type (Figure 1).

#### 3.4.1 Day 1

##### 3.4.1.1 Water samples

- Mix the sample by inverting the bottle.
- Prefilter the sample through a 10 μm pore-size filter and collect the flow-through.
- Filter approximately 100 mL through a 0.45 μm pore-size filter and transfer the filter to a sterile petri dish.
- Cut the filter into three pieces with a sterile scalpel and transfer each filter piece to 5 mL of TSB, EE, and SB medium, respectively.
- Incubate the samples overnight at 37 °C at 180 rpm shaking. When a turbidity of 4.5–6.0 McFarland is reached, a biobank sample of the culture is taken as described above.

##### 3.4.1.2 Meat samples

- Check whether the cold chain was maintained during transport of meat samples.
- Open the meat package under sterile conditions.
- Take sterile tweezers and a sterile scalpel and cut out small pieces (approximately 0.5 × 0.5 cm) of the meat at various points for homogenization. Inoculate 5 mL of TSB, EE, SB, and GC medium with 100 μL of the homogenate, respectively.
- Incubate the samples overnight at 37 °C at 180 rpm shaking. When a turbidity of 4.5–6.0 McFarland is reached, a biobank sample of the culture is taken as described above.

##### 3.4.1.3 Soil samples

- Resuspend a spatula tip of soil in 5 mL each of TSB, EE, and GC medium to form a homogeneous suspension.
- Incubate the samples overnight at 37 °C at 180 rpm shaking. When a turbidity of 4.5–6.0 McFarland is reached, a biobank sample of the culture is taken as described above.

##### 3.4.1.4 Fly samples

- Transfer a fly to a 1.5 mL tube containing 300 μL of TSB medium.
- Homogenize the fly using a sterile pestle.
- All samples originating from risk areas where *Bacillus anthracis* or *Bacillus cereus* biovar *anthracis* may be present shall be subjected to qPCR analysis prior to any cultivation procedures (Hoffmann et al., 2017). Samples yielding positive qPCR results shall not be cultivated.
- Add additional 700 μL of TSB medium and mix thoroughly.
- Inoculate 5 mL of TSB, EE, and GC medium with 100 μL of fly homogenate.
- Incubate the samples overnight at 37 °C at 180 rpm shaking. When a turbidity of 4.5–6.0 McFarland is reached, a biobank sample of the culture is taken as described above.
- Centrifuge the remaining sample for 2 min at 13,000 rpm and discard the supernatant.
- Store the pellet at −70 °C or −80 °C for further analysis (i.e., DNA extraction for metagenomics).

##### 3.4.1.5 Swab samples

Swab samples can be taken in either semi-solid Amies agar or liquid Amies medium. The initial processing differs as follows:

• Semi-solid Amies agar swabs.
  ○ Cut the swab into a 2 mL tube containing 750 μL TSB medium.
  ○ Vortex the sample thoroughly to resuspend bacteria from the swab.
  ○ Transfer the swab to a new sterile tube.
  ○ Fill the bacterial suspension up to 1.5 mL TSB.
• eSwab with liquid Amies medium.
  ○ Vortex the eSwab thoroughly to resuspend bacteria from the swab.
  ○ Transfer the swab to a new sterile tube.
• From now on, both swab samples will be processed in the same way.
  ○ Use 100 μL bacterial suspension to inoculate 5 mL of TSB, EE, SB, and GC medium.
  ○ Incubate the samples overnight at 37 °C at 180 rpm shaking. When a turbidity of 4.5–6.0 McFarland is reached, a biobank sample of the culture is taken as described above.
  ○ If no eNAT swab for metagenomics was taken: centrifuge the remaining sample for two minutes at 13,000 rpm, discard the supernatant and add the respective swab to the pellet.
  ○ Store the pellet at −70 °C or −80 °C for further analysis (DNA extraction for metagenomics).

#### 3.4.2 Day 2

Subsequent processing steps are performed uniformly across all sample types.

• Spread the overnight cultures onto the following agar plates according to the scheme in Figure 2. In short: Spread 20 μL of bacterial culture evenly on 1/4 of the agar plate. Make two isolation smears starting in the quarter where the culture was spread.
  ○ TSB culture is spread on CHROMagar™ mSuperCARBA.
  ○ EE culture is spread on CHROMagar™ ESBL.
  ○ GC culture is spread on CHROMagar™ MRSA.
  ○ SB culture is spread on XLD agar.
• Incubate the agar plates overnight at 37 °C. CHROMagar™ MRSA should be cultured anaerobically for specific enrichment of MRSA, such as anaerobic jars. This step prevents the growth of microorganisms such as micrococci and enhances the growth of MRSA.

**FIGURE 2:**
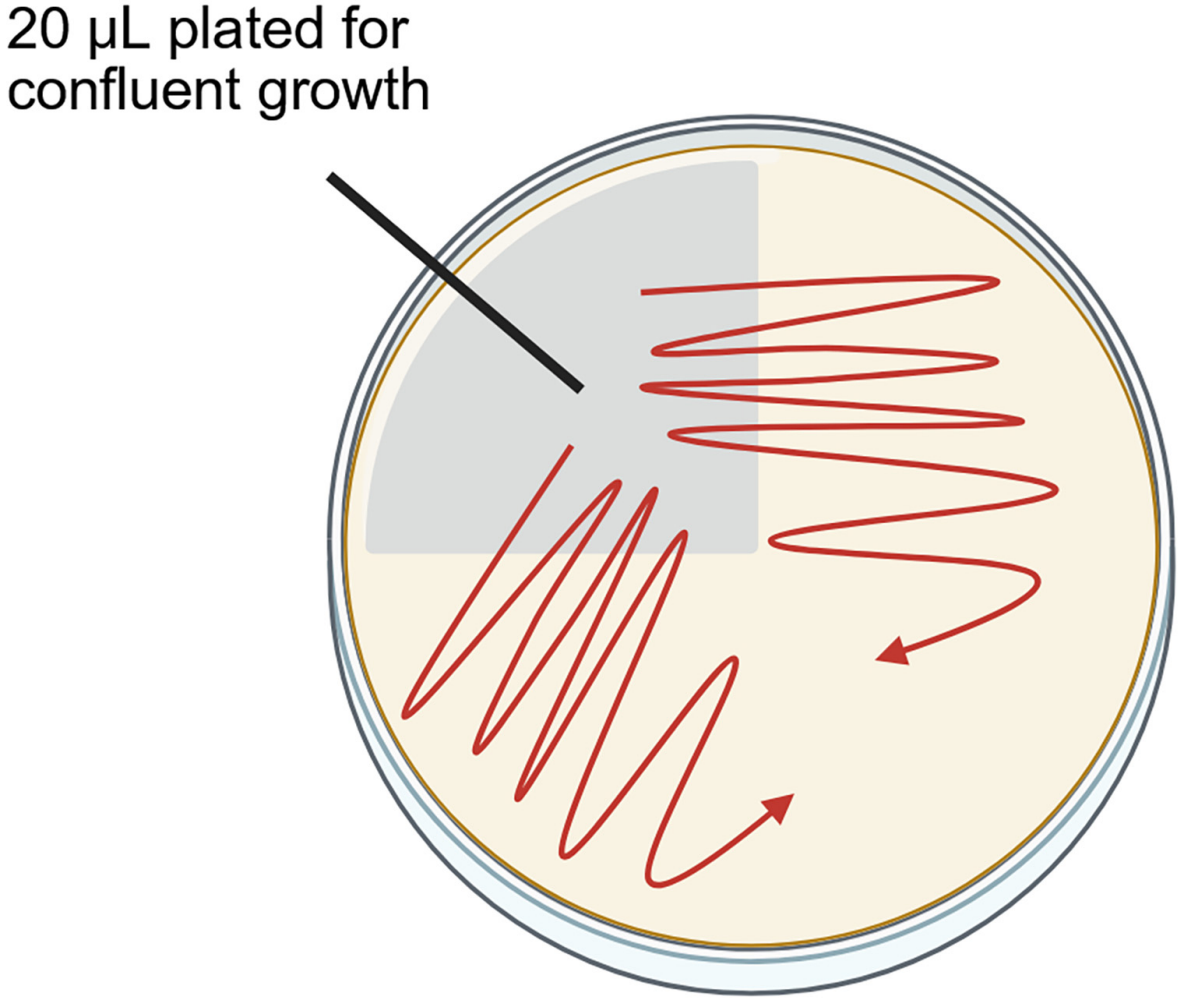
Spread of the overnight culture on an agar plate for better isolation of bacteria. Created in BioRender. Bahr, A. (2026) https://BioRender.com/6926uz2.

#### 3.4.3 Day 3

- Growth on CHROMagar™ MRSA: Rose to mauve colonies indicate the growth of MRSA. Use one colony for an isolation streak on a new CHROMagar™ MRSA plate to obtain a pure culture. Incubate the agar plates overnight at 37 °C anaerobically, e.g., in anaerobic jars.

- Growth on XLD agar: Black colonies indicate the growth of *Salmonella* spp. Use one black colony for an isolation streak on a new XLD agar plate to obtain a pure culture. Incubate the agar plates overnight at 37 °C.

Different bacterial species form different colony phenotypes on chromogenic agar. *Escherichia coli*, for example, forms red colonies, while bacteria from the KEC group (*Klebsiella* spp.*, Enterobacter* spp. and *Citrobacter* spp.) present as blue colonies on CHROMagar™ mSuperCARBA and CHROMagar™ ESBL. *Acinetobacter* spp. and *Pseudomonas* spp. show white colonies. To isolate bacteria with different resistance patterns, eight colonies per phenotype are picked.

• Take the respective agar plates and place them on the “picker grid” (template dividing an agar plate into numbered sectors). Mark the agar plates in the same place to always ensure the orientation of the agar plate. The order of the agar plates varies depending on the phenotype and the plate on which the bacteria grew (Figure 3). The order is as follows:
  ○ Colored colonies from CHROMagar™ ESBL to:
    1. CHROMagar™ ESBL
    2. CHROMagar™ Serratia (only for wildlife or environmental samples)
    3. CHROMagar™ mSuperCARBA
    4. CHROMagar™ Orientation supplemented with ciprofloxacin (1 mg/mL)
  ○ Colored colonies from CHROMagar™ mSuperCARBA to:
    1. CHROMagar™ mSuperCARBA
    2. CHROMagar™ Serratia (only for wildlife or environmental samples)
    3. CHROMagar™ ESBL
    4. CHROMagar™ Orientation supplemented with ciprofloxacin (1 mg/mL)
  ○ White colonies from CHROMagar™ ESBL to:
    1. CHROMagar™ Acinetobacter
    2. CHROMagar™ mSuperCARBA
  ○ White colonies from CHROMagar™ mSuperCARBA to:
    1. CHROMagar™ mSuperCARBA
    2. CHROMagar™ Acinetobacter
• Select a well-isolated colony using a sterile toothpick and gently inoculate the corresponding numbered field on the respective agar plates (Figure 3). Use minimal biomass to avoid false-positive growth and ensure reliable result interpretation.
• Select eight colonies in total of the same phenotype for picking. If fewer than eight colonies of a given phenotype were present, all available colonies of that phenotype were picked.
• Incubate the CHROMagar™ Acinetobacter plates overnight at 42 °C and all other plates at 37 °C.

**FIGURE 3:**
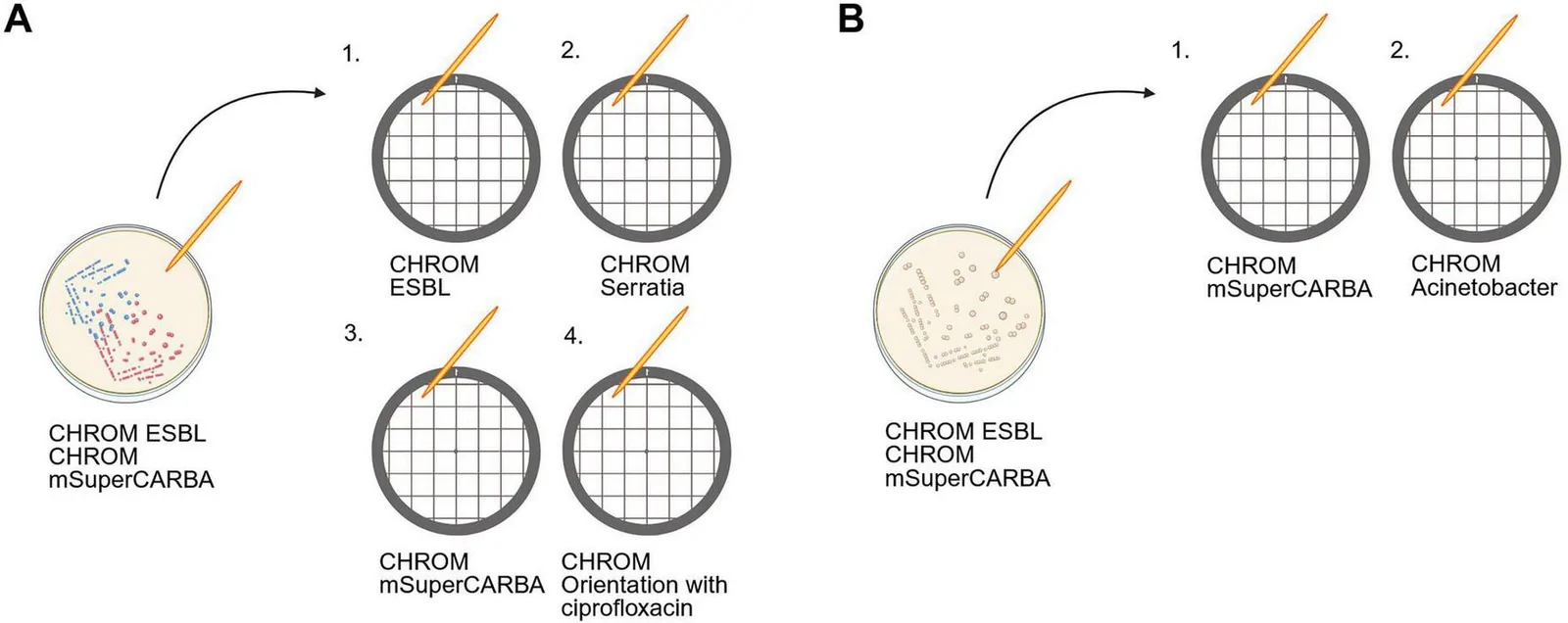
Schematic representation of the picking scheme and the order of selective CHROMagar™ plates for panels **(A)** colored colonies and **(B)** white colonies. From the initial agar plate, which was incubated overnight at 37 °C, a colony is picked up with a toothpick and gently pressed onto the same position in the picking grid. ESBL: extended-spectrum beta-lactamase. Created in BioRender. Bahr, A. (2026) https://BioRender.com/zv7u98s.

#### 3.4.4 Day 4

• Growth on XLD agar: Take a well-separated colony from the first isolation streak and spread the colony on a new agar plate. The same applies to MRSA agar.
• Growth on CHROMagar™ Acinetobacter: Compare growth on CHROMagar™ Acinetobacter and CHROMagar™ mSuperCARBA. Only colonies that have grown on both plates will be included in *Acinetobacter*-specific *gyrB* multiplex colony PCR according to Higgins et al. (2010).
• Growth on picker plates:
  ○ Place the corresponding agar plates from the picker scheme back on the picker grid.
  ○ Compare the growth of the colonies in the fields with the same numbers.
  Colonies that show different growth patterns are selected according to Tables 2, 3.
  ○ Take one colony per phenotype for each different resistance pattern and isolate the colony on a new CHROMagar™ plate with the corresponding selection pressure. ESBL-positive colonies are further isolated on a CHROMagar™ ESBL plate. However, once a colony has grown on CHROMagar™ mSuperCARBA, this agar continues to be used.
• Incubate the agar plates overnight at 37 °C.

**TABLE 2:** Interpretation of growth of colored colonies on the picking plates.

Colony color	Growth on CHROMagar™ ESBL	Growth on CHROMagar™ mSuperCARBA	Growth on CHROMagar™ with ciprofloxacin	Growth on CHROMagar™ Serratia	Interpretation
Blue or reddish blue	+	−	−	−	ESBL-positive KEC → further analysis
Blue or reddish blue	+	−	−	+	See note 1 in caption
Blue or reddish blue	+	+	−	−	ESBL-positive + carbapenem-resistant KEC → further analysis
Blue or reddish blue	+	+	−	+	See note 1 in caption
Blue or reddish blue	+	+	+	−	MDR KEC → further analysis
Blue or reddish blue	+	+	+	+	See note 1 in caption
Blue or reddish blue	−	+	−	−	Carbapenem-resistant KEC → further analysis
Blue or reddish blue	−	+	−	+	See note 1 in caption
Blue or reddish blue	+	−	+	−	ESBL-positive + ciprofloxacin resistant KEC → further analysis
Blue or reddish blue	+	−	+	+	See note 1 in caption
Blue or reddish blue	−	+	+	−	Carbapenem- and ciprofloxacin-resistant KEC → further analysis
Blue or reddish blue	−	+	+	+	See note 1 in caption
Red	+	−	−	−	ESBL-positive *E. coli* → further analysis
Red	+	−	−	+	See note 1 in caption
Red	+	+	−	−	ESBL-positive + carbapenem-resistant *E. coli* → further analysis
Red	+	+	−	+	See note 1 in caption
Red	+	+	+	−	MDR *E. coli* → further analysis
Red	+	+	+	+	See note 1 in caption
Red	−	+	−	−	Carbapenem-resistant *E. coli* → further analysis
Red	−	+	−	+	See note 1 in caption
Red	+	−	+	−	ESBL-positive + ciprofloxacin resistant *E. coli* → further analysis
Red	+	−	+	+	See note 1 in caption
Red	−	+	+	−	Carbapenem- and ciprofloxacin resistant *E. coli* → further analysis
Red	−	+	+	+	See note 1 in caption

On the WHO’s list of priority bacterial pathogens, *Enterobacterales* that are resistant to carbapenems and third-generation cephalosporins are classified as a “critical group”. Therefore, colonies are selected in the screening protocol based on these resistances. Ciprofloxacin was chosen to cover a third class of antibiotics, thereby providing more criteria for selecting colonies based on their resistance patterns. Colonies that are resistant only to ciprofloxacin do not occur, since screening for ESBL or carbapenem resistance was performed beforehand, ensuring that growth on these plates is present. Only colonies that are resistant to all three classes of antibiotics are considered multidrug-resistant. CHROMagar™ is designed to differentiate different bacterial species by colony color. *E. coli* colonies show red colonies, *Klebsiella* spp., *Enterobacter* spp., *Citrobacter* spp. appear as blue colonies. “+” is defined as colony growth, while “−” means no growth; ESBL: extended-spectrum beta-lactamase; KEC: *Klebsiella* spp., *Enterobacter* spp., *Citrobacter* spp.; MDR: multi-drug-resistant; Note 1: There will be three different phenotypes on Serratia agar: metallic blue/blue-green colonies indicating *Serratia* spp. (other than *Serratia marcescens*) and reddish blue colonies indicating *Citrobacter* spp. Red colonies indicate the growth of a putative *Serratia marcescens*. Other bacterial species will not grow on Serratia agar. In case you have a reddish blue or red colony on Serratia agar check if there is additional growth on ESBL or carbapenem agar. If yes, use this colony for further analysis. If not, no further analysis.

**TABLE 3:** Interpretation of growth of white colonies on the picking plates.

Colony color	Growth on CHROMagar™ Acinetobacter	Growth on CHROMagar™ mSuperCARBA	Interpretation
White	−	−	No *Pseudomonadales* no → further analysis
White	+	−	No carbapenem-resistant *Pseudomonadales* no → further analysis
White	−	+	No carbapenem-resistant *Pseudomonadales* no → further analysis
White	+	+	Carbapenem-resistant *Pseudomonadales* → further analysis

*Pseudomonadales* spp. will grow as white colonies on CHROMagar™ mSuperCARBA and as red colonies on CHROMagar™ Acinetobacter. “+” is defined as colony growth, while “−” means no growth.

#### 3.4.5 Day 5

- Store XLD and MRSA plates in the fridge and continue on day 6.
- For the other bacteria: Take a well-separated colony from the first isolation streak and spread the colony on a new agar plate.
- Incubate the plates overnight at 37°C.

#### 3.4.6 Day 6

- Take a well-separated colony from the second isolation streak and spread the colony on a TSA plate for VITEK (or alternative) species identification.
- Incubate the agar plates overnight at 37 °C.

#### 3.4.7 Day 7

- Use the VITEK2 system, MALDI-TOF, or an alternative species identification method according to the manufacturer’s instructions. In laboratories with limited equipment availability, particularly in low- and middle-income countries (LMICs), commercially available biochemical identification systems (e.g., API strips, bioMérieux, France) may provide a suitable alternative. In this protocol, VITEK2 GN cards for Gram-negative bacteria and VITEK2 GP cards for Gram-positive bacteria (bioMérieux, France) were used.
- Once a bacterium of interest has been identified, antimicrobial susceptibility testing can also be performed using VITEK2. The VITEK2 AST N428 (bioMérieux, France) card was used in this study for AST of gram-negative bacteria. The AST results should be interpreted in accordance with the breakpoints set out by the European Committee on Antimicrobial Susceptibility Testing (EUCAST) or other relevant guidelines.

#### 3.4.8 Day 8

- Take one colony from the TSA plate to inoculate 5 mL of TSB with the respective selection pressure. Incubate the culture at 37 °C and 180 rpm shaking.
- When the culture has reached a turbidity increase of 4.5–6.0 McFarland then 800 μL of the culture is sterilely removed and transferred to a sterile cryotube containing 200 μL of glycerol. The sample is thoroughly mixed by pipetting up and down and stored directly at −70 °C or −80 °C.

### 3.5 Bacterial DNA isolation and whole genome sequencing

For downstream analyses and verification, total DNA was extracted from all isolates using the MasterPure™ DNA Purification Kit for Blood Version II (Lucigen, Middleton, WI, USA), according to the manufacturer’s instructions, or the Monarch Spin gDNA Extraction Kit (New England Biolabs, Ipswich, MA, USA) according to the manufacturer’s instructions. DNA was then quantified fluorometrically using the Qubit 4 Fluorometer and the corresponding dsDNA BR Assay Kit (Thermo Fisher Scientific, Waltham, MA, USA). DNA of the fly isolates was sequenced by SeqCenter (Pittsburgh, PA, USA) using Illumina sequencing by synthesis (SBS) technology. The sequencing libraries were prepared using the tagmentation-based and PCR-based Illumina DNA Prep kit and custom IDT 10 bp unique dual indices (UDI) with a target insert size of 280 bp. No additional DNA fragmentation or size selection steps were performed and sequencing was performed on an Illumina NovaSeq X Plus sequencer in one or more multiplexed shared-flow-cell runs, producing 2 × 151 bp paired-end reads. Demultiplexing, initial quality control and initial adapter trimming was performed with bcl-convert (v4.2.4). DNA of all other isolates was sequenced by Novogene (Cambridge, UK) also using Illumina SBS technology on a NovaSeq X Plus sequencer.

### 3.6 Sequence assembly and genomic analyses

Raw paired-end sequencing reads were contaminant-filtered, adapter-trimmed and quality-trimmed using BBDuK from BBTools [fly isolates: v39.28; waste water and surface swab isolates: v39.30; all other isolates: v39.34^1^, (Accessed April 12. 2026)]. Quality control of raw and trimmed reads was done with FastQC [v0.12.1^2^, (Accessed April 12. 2026)]. Assemblies were created using shovill [v1.1.0^3^, (Accessed April 12. 2026)] in combination with SPAdes [v4.2.0, (Prjibelski et al., 2020)] at a maximum sequence coverage of 100x without using the integrated correction step. A manual polishing step was performed by mapping all trimmed paired-end reads to the draft assembly using BWA-MEM2 [fly isolates: v2.2.1; all other isolates: v2.3, (Vasimuddin et al., 2019)] and polishing the assembly with Polypolish [fly, waste water and surface swab isolates: v0.6.0; all other isolates: v0.6.1, (Wick and Holt, 2022)]. Multilocus Sequence Typing (MLST) was done with mlst [fly, waste water and surface swab isolates: v2.23.0; all other isolates: v2.32.2^4^, (Accessed April 12. 2026)] using the corresponding schemes hosted at the PubMLST (Jolley et al., 2018) and BIGSdb-Pasteur websites. Antimicrobial resistance and stress genes as well as virulence features were predicted with AMRFinderPlus [fly, waste water and surface swab isolates: v4.0.23 with DB v2025-06-03.1; all other isolates: v4.2.7 with DB v2026-01-21.1 (Feldgarden et al., 2021)].

## 4 Results and discussion: Demonstration and evaluation of the workflow

### 4.1 Diversity and complexity of the sample material

This study aimed to establish a simple protocol for processing diverse One Health samples to ensure methodological comparability. Most previous studies have focused on single sample types (e.g., water or soil) (Mahmud et al., 2023; Armalytė et al., 2019). To our knowledge, the protocol presented here represents a novel, cross-matrix approach within One Health research. A uniform processing workflow is essential to ensure qualitative comparability of bacterial isolates across different sample types, thereby allowing observed differences in species diversity to be attributed to the sample origin and complexity rather than to methodological bias. In addition, this study aimed to isolate and identify multiple ARB, including phenotypically similar isolates, as several strains of the same species may coexist within a single sample. The objective was not to comprehensively culture all bacteria (culturomics), but to capture representative microbial diversity. To demonstrate and establish the applicability of the protocol across sample types of varying complexity, we applied it to agricultural soil and poultry meat from Germany, fecal swabs from wild birds in Germany, hospital surface swabs and waste water from Côte d’Ivoire, and flies collected in Taï National Park (Côte d’Ivoire) (Table 1). These samples were part of cohort research projects in collaboration with international partners. Several hundred samples were processed according to this protocol for the development and validation of the method.

### 4.2 Isolation of selected AMR bacteria within the picker scheme

The picker scheme is designed to preferentially isolate bacterial species classified as critical priority pathogens by the World Health Organization, including third-generation cephalosporin-resistant and carbapenem-resistant *Enterobacterales* as well as carbapenem-resistant *Acinetobacter baumannii* (WHO, 2024). These pathogens cause severe infections and exhibit resistance to last-resort antibiotics, substantially limiting treatment options. Their pronounced ability to acquire and disseminate resistance determinants, persist in clinical and environmental settings, and circulate across human, animal, and environmental reservoirs makes them particularly relevant One Health pathogens (García-González et al., 2025; Guido et al., 2025). Targeted isolation and characterization of these bacteria from diverse One Health samples are essential to elucidate transmission pathways, enable early detection of emerging resistance mechanisms, and support effective prevention and control strategies. The selective enrichment broths and corresponding CHROMagar™ plates used in this protocol facilitate preferential growth of these target organisms. Consequently, other ARB may be present in the samples but are either not sufficiently enriched for isolation or are suppressed by the selective antibiotics applied. A key strength of the picker scheme is its flexibility, as it can be readily adapted to target additional pathogens by modifying the selective media and antibiotic composition. For the processing of water samples, reinforced clostridial medium can be used to enrich *Clostridium* spp., followed by cultivation on appropriate chromogenic agar (Hafiz and Oakley, 1976). Similarly, animal-derived samples can be processed using Bolton broth in combination with CCDA agar for the selective enrichment and detection of *Campylobacter* spp. (Seliwiorstow et al., 2016). In many previous One Health surveillance studies, only a single or a few bacterial colonies per phenotype and resistance profile were selected for further analysis (Dahms et al., 2015; Lechleiter et al., 2025). However, multiple ARB of the same species may coexist within a single sample. For example, incubation of a sample on CHROMagar™ ESBL may yield several pink colonies indicative of *Escherichia coli*. Although all colonies are ESBL-positive, they may differ in their resistance to other antibiotic classes or may represent identical strains. This protocol is designed to detect such intra-species diversity by enabling the isolation of multiple phenotypically distinct isolates of the same species. Single colonies are picked onto agar plates containing ESBL reagents, carbapenems, and ciprofloxacin, allowing direct comparison of growth patterns and the identification of isolates with differing antibiotic resistance profiles. Within the picker scheme, eight colonies per phenotype and sample are selected. This number reflects the binary outcomes of growth versus no growth across three selection markers (carbapenem, ESBL and ciprofloxacin), resulting in eight (2^3^) possible phenotypic combinations per phenotype. Interpretation of these combinations is provided in Tables 2, 3 and is based on the WHO bacterial priority pathogen list. Based on the phenotypic profiles obtained, selected colonies are subjected to further downstream analyses. *Serratia* species are ubiquitous and occur in intestinal microbiota as well as in soil, water, and plants (Williams et al., 2022). Because *Serratia* spp. produce colonies with the same color as the KEC group on CHROMagar™, differentiation can be challenging. Therefore, Serratia agar is applied to wildlife and environmental samples to exclude *Serratia* spp. and enable early identification of false-positive colonies rather than for targeted isolation. An exception is the appearance of red colonies, which indicates prodigiosin producing species like the clinically relevant pathogen *Serratia marcescens*. According to the manufacturer’s specifications, CHROMagar™ Acinetobacter is designed to selectively support the growth of *Acinetobacter* spp., which typically form red colonies. However, previous studies have demonstrated that non-*Acinetobacter* species may also produce red colonies on these media (Ajao et al., 2011). To address this limitation, the workflow incorporates preselection on carbapenem-containing agar, which may yield *Acinetobacter* spp. as well as other carbapenem-tolerant non-fermenters, including *Pseudomonas* spp. and *Stenotrophomonas maltophilia*. Incubation at an elevated temperature (42 °C instead of 37 °C) is included as an additional selective step to suppress the growth of environmentally abundant species such as *Pseudomonas putida*, while increasing the likelihood of isolating members of the *Acinetobacter calcoaceticus–baumannii* complex (Oberhofer, 1979). To enable reliable discrimination of *Acinetobacter* spp. from other bacteria forming red colonies, a colony PCR based on the *gyrB* multiplex assay described by Higgins et al. (2010) was applied, allowing species-level differentiation within the *A. calcoaceticus–baumannii* complex and clear separation from non-*Acinetobacter* taxa. For each sample, one colony displaying an *A. baumannii*-specific banding pattern and one colony lacking a *gyrB* amplification signal were selected for further processing. In combination with the picker scheme and subsequent MALDI-TOF MS analysis, this approach enables the parallel and reliable isolation and identification of *Acinetobacter* and *Pseudomonas* species from complex samples.

### 4.3 Demonstration and evaluation of workflow performance

Application of the workflow to samples of differing complexity resulted in variable numbers and compositions of bacterial isolates, reflecting differences in sample matrix complexity rather than protocol performance (Figure 4 and Supplementary Table 1). The complexity of the samples in terms of AMR abundance and relevance shows that samples with high relevance (waste water, surface swabs) exhibit a higher number of isolates than those with low relevance (wild birds and flies). For example, application of the method to fly samples collected in Taï National Park resulted in one to two *E. coli* isolates per sample. From fly 1, a single *E. coli* isolate (GEAR5612) was recovered, whereas two *E. coli* isolates were obtained from fly 2 and the wild bird samples. In contrast, more complex matrices yielded a higher diversity of isolates. Three distinct bacterial species were recovered from the meat sample, while five different bacteria were isolated from the soil sample. *Serratia marcescens* (GEAR7374) was successfully isolated from soil using Serratia-selective agar and has therefore no growth in Figure 4 on the other selective agars. In addition, one *Enterobacter ludwigii* (GEAR7376) isolate was recovered despite the absence of phenotypic resistance, consistent with the intrinsic resistance (*bla*_ACT_) of *Enterobacter* spp. to 3^rd^ generation cephalosporins. Application of the workflow to waste water samples yielded five and seven distinct bacterial isolates, respectively. Although *E. coli* and *Klebsiella pneumoniae* were repeatedly recovered, isolates from the same sample belonged to different sequence types, demonstrating the ability of the protocol to resolve within-sample diversity. Application of the picker scheme to waste water sample 1 enabled the isolation of three distinct *K. pneumoniae* isolates (GEAR6136, GEAR6137, GEAR6140). Each isolate displayed a distinct picking pattern, which was reflected in differences in phenotypic resistance profiles. WGS confirmed that the three isolates belonged to different sequence types, demonstrating that the picker scheme can resolve multiple strains of the same species within a single complex sample (Supplementary Table 1). Similarly, application of the workflow to fly sample 2 resulted in the isolation of two *E. coli* isolates with distinct phenotypic and genotypic profiles. In waste water sample 2, four *E. coli* isolates were recovered. Two isolates displayed identical picker patterns and would therefore typically be represented by a single isolate in downstream analyses. However, visible differences in colony morphology across all picker plates prompted inclusion of additional isolates. Subsequent WGS demonstrated that these isolates belonged to different sequence types, illustrating that incorporation of colony morphology can further enhance strain resolution beyond phenotypic picker patterns alone. In contrast, three *E. coli* isolates recovered from waste water sample 1 included two isolates with identical picker patterns but differing colony morphologies. WGS and single-nucleotide polymorphism analysis revealed that these two isolates belonged to the same sequence type and were clonal. This example illustrates that genetically identical clones may occasionally be isolated despite phenotypic or morphological variation, and that definitive discrimination between identical and distinct isolates ultimately requires genomic analysis. Such cases were rare across several hundred samples processed using this workflow and do not outweigh the overall advantages of the method. Application of the picker scheme to a surface swab sample yielded three *K. pneumoniae* isolates with distinct picker patterns (Figure 4). AST showed largely similar resistance profiles, with GEAR5862 and GEAR5864 differing only in imipenem resistance. WGS confirmed that all three isolates belonged to different sequence types (Supplementary Table 1). This example demonstrates that the picker scheme can resolve genetically distinct strains even when phenotypic resistance profiles are highly similar. Growth patterns on selective picker plates may be influenced by multiple factors. In this example, although all three *K. pneumoniae* isolates displayed phenotypic carbapenem resistance, only one isolate grew on CHROMagar™ mSuperCARBA. As the precise composition and antibiotic concentrations of selective supplements are proprietary, potential explanations include local variability in antibiotic concentration during plate preparation or the presence of resistance in a subpopulation (heteroresistance) (El-Halfawy and Valvano, 2015). Notably, none of the isolates carried known carbapenem resistance genes, suggesting resistance mechanisms such as efflux pump activity or porin alterations (Mammeri et al., 2025). Such mechanisms may be detected by broth-based susceptibility testing but may not consistently support growth on carbapenem-containing agar. Therefore, positive and negative controls can be used to exclude false-positive colonies. In addition to the *K. pneumoniae* isolates, one *E. coli* isolate was recovered from the same surface swab sample. Like all culture-based workflows, this protocol is subject to biases introduced by selective enrichment and cultivation conditions, which may favor the recovery of specific bacterial taxa while underrepresenting others. Furthermore, non-culturable or viable but non-culturable microorganisms cannot be recovered using this approach. Nevertheless, in the context of One Health surveillance, cultivation-based approaches offer distinct advantages over metagenomic methods, particularly for monitoring AMR across heterogeneous sample types from humans, animals, food, and the environment. While metagenomics enables culture-independent detection of resistance genes, its applicability is often limited by variable microbial biomass, host DNA contamination, and difficulties in reliably linking resistance determinants to specific bacterial hosts in complex matrices such as waste water, soil, or livestock samples (Quince et al., 2017; Hendriksen et al., 2019). In addition, although metagenomic data can reveal resistance signatures and population-level trends, robust identification of high-risk clonal lineages and strain-level transmission dynamics remains challenging and strongly depends on sequencing depth, reference genome availability, and advanced bioinformatic pipelines, limiting its routine use for actionable surveillance (Djordjevic et al., 2024). Cultivation-based surveillance enables the isolation and in-depth characterization of viable ARB, allowing phenotypic resistance profiling, clonal lineage assignment, and direct comparison across sectors, thereby facilitating the detection of high-risk clones and transmission pathways within the One Health continuum (Berendonk et al., 2015). Importantly, in low- and middle-income countries, where access to high-throughput sequencing infrastructure and bioinformatics expertise is often limited, culture-based approaches remain more feasible, cost-effective, and sustainable for long-term AMR surveillance (Aruhomukama, 2022). For longitudinal surveillance, a cultivation approach is better suited to identifying identical pathogens over a longer period of time; this is not possible with metagenomics alone (Olsen and Riber, 2025). Integrating cultivation with metagenomic approaches can further strengthen One Health surveillance by combining comprehensive resistome profiling with isolate-level resolution. This integration allows resistance genes detected in complex microbial communities to be linked to specific bacterial hosts and subsequently validated through phenotypic analyses (Yamin et al., 2023). Such validation is essential, as metagenomic predictions alone do not necessarily correspond to expressed or clinically relevant resistance phenotypes, and experimental confirmation is required to distinguish functional resistance determinants from non-functional or context-dependent gene variants (Boolchandani et al., 2019). Overall, integrative strategies enhance the sensitivity, interpretability, and actionability of surveillance data across sectors and provide a robust and scalable framework for global AMR monitoring (Wright, 2010; Tacconelli et al., 2018).

**FIGURE 4:**
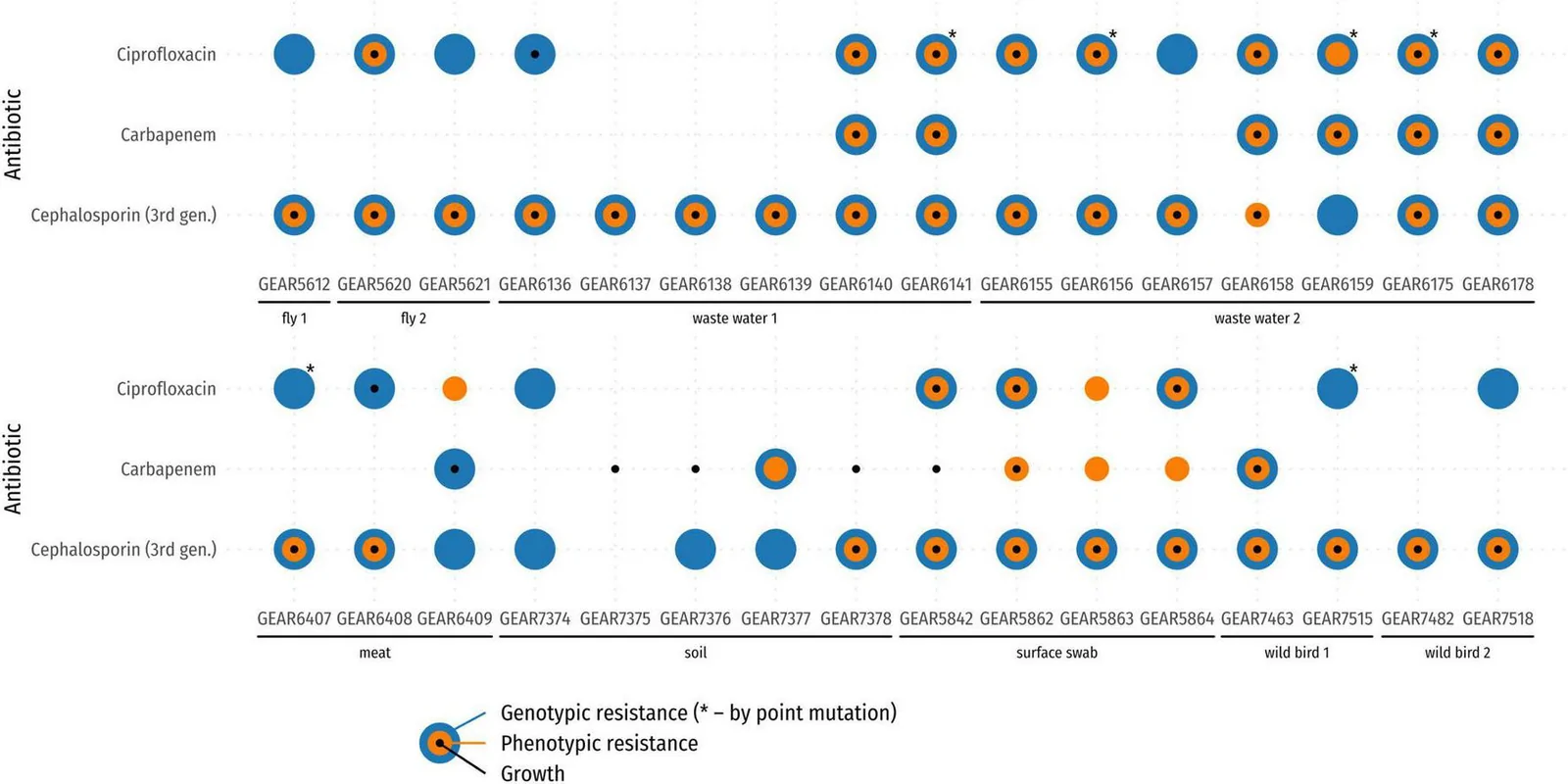
Phenotypic and genotypic resistance profiles of isolates across diverse One Health sample types. Overview of growth on selective agar plates and corresponding phenotypic and genotypic antimicrobial resistance profiles of bacterial isolates recovered from different One Health sample matrices, including flies, waste water, meat, soil, surface swabs, and wild birds. Each column represents an individual isolate, grouped by sample origin, while rows indicate the tested antibiotic classes (ciprofloxacin, carbapenems, and third-generation cephalosporins). The black dots indicate growth on selective media containing the respective antibiotics. Phenotypic resistance is indicated by orange circles and genotypic resistance determinants identified by whole-genome sequencing are represented by blue circles; asterisks (*) indicate resistance associated with point mutations.

## 5 Troubleshooting

### 5.1 No individual colonies after spreading the liquid culture

This may occur if agar plates are still moist, causing condensation and uncontrolled spreading of the bacterial suspension. In addition, failure to use a new or flame-sterilized inoculation loop between smears, or placing smears too close together, can prevent adequate spatial separation of colonies. Solution: Re-isolate bacteria by picking material from three distinct locations on the agar plate and repeat isolation on a fresh plate following the smear scheme shown in Figure 2. Incubate overnight at 37 °C.

### 5.2 Colonies show a different color on picker plates than on the original plate

This may indicate the presence of a mixed culture. For example, a red colony on an ESBL plate may retain this phenotype on ESBL agar but appear blue on ciprofloxacin-containing agar, reflecting the presence of two subpopulations with different resistance profiles. Solution: In such cases, growth should be interpreted independently for each selective medium. The red colony should be scored as ESBL-positive only. If no blue colonies were initially selected, or if blue colonies fail to grow on ciprofloxacin-containing agar, the blue colony phenotype may be included in subsequent analyses.

### 5.3 No growth on the agar plate after the first isolation smear

Lack of growth after initial isolation may result from a false-positive pick caused by excessive transfer of cell material, leading to artificial growth signals not associated with true resistance. Solution: Minimize the amount of biomass transferred during picking and ensure that growth on picker plates appears as discrete, raised colonies extending beyond the application site.

### 5.4 Interpretation of growth on Serratia agar

On Serratia agar, metallic blue to blue-green colonies were interpreted as *Serratia* spp. (excluding *S. marcescens*), reddish-blue colonies as *Citrobacter* spp., and red colonies as presumptive *S. marcescens*. For reddish-blue or red colonies, growth on ESBL agar or carbapenem agar was assessed. Colonies showing corresponding growth on either medium were selected for further analysis, whereas colonies without growth on either medium were not analyzed further.

## 6 Conclusion

This workflow enables the generation of directly comparable datasets from heterogeneous One Health sample sources. It is suitable for both high-throughput laboratory settings and resource-limited field laboratories and can be readily adapted to a wide range of surveillance, research, and intervention study designs. Its scalability, adaptability, and cross-matrix applicability make it a valuable resource for the global AMR research community. Application of the workflow across sample types of varying complexity demonstrates its capacity to isolate multiple bacterial species and distinct strains, including within-species diversity, prior to downstream analyses such as whole-genome sequencing. Importantly, the protocol supports unified sample processing across matrices, thereby enabling qualitative comparability of AMR surveillance data and facilitating robust monitoring of AMR circulation and spread across the One Health continuum. Although the method is more time-intensive than conventional approaches, this represents a deliberate and advantageous trade-off, as increased processing depth directly translates into higher surveillance sensitivity. By systematically capturing a substantially broader spectrum of distinct isolates—including co-occurring strains of the same species—the workflow maximizes the probability of detecting clinically and epidemiologically relevant diversity, thereby significantly increasing the informational yield per sample and reducing the risk of underestimating AMR complexity.

## Data Availability

The data analyzed in this study is subject to the following licenses/restrictions: the datasets used in this study were employed solely for the purpose of demonstrating and explaining the applied methodology. They are therefore not publicly available at this time. The data can be made available by the authors upon reasonable request. In addition, the datasets are planned to be deposited in a public repository at a later stage together with other related studies from which the data originate. Requests to access these datasets should be directed to the corresponding author.

## Footnotes

1
https://bbmap.org

2
https://www.bioinformatics.babraham.ac.uk/projects/fastqc

3
https://github.com/tseemann/shovill

4
https://github.com/tseemann/mlst

## References

[B1] Ajao A. O. Robinson G. Lee M. S. Ranke T. D. Venezia R. A. Furuno J. P. et al. (2011). Comparison of culture media for detection of *Acinetobacter baumannii* in surveillance cultures of critically-ill patients. *Eur. J. Clin. Microbiol. Infect. Dis*. 30, 1425–1430. 10.1007/s10096-011-1237-7 21487763 PMC3660032

[B2] Armalytė J. Skerniškytė J. Bakienė E. Krasauskas R. Šiugždinienė R. Kareivienė V. (2019). Microbial diversity and antimicrobial resistance profile in microbiota from soils of conventional and organic farming systems. *Front. Microbiol*. 10:892. 10.3389/fmicb.2019.00892 31105678 PMC6498881

[B3] Aruhomukama D. (2022). Antimicrobial resistance data, frugal sequencing, and low-income countries in Africa. *Lancet Infect. Dis*. 22, 933–934. 10.1016/S1473-3099(22)00312-7 35594898

[B4] Berendonk T. U. Manaia C. M. Merlin C. Fatta-Kassinos D. Cytryn E. Walsh F. et al. (2015). Tackling antibiotic resistance: The environmental framework. *Nat. Rev. Microbiol*. 13, 310–317. 10.1038/nrmicro3439 25817583

[B5] Boolchandani M. D’Souza A. W. Dantas G. (2019). Sequencing-based methods and resources to study antimicrobial resistance. *Nat. Rev. Genet*. 20, 356–370. 10.1038/s41576-019-0108-4 30886350 PMC6525649

[B6] Dahms C. Hübner N. O. Kossow A. Mellmann A. Dittmann K. Kramer A. (2015). Occurrence of ESBL-producing *Escherichia coli* in livestock and farm workers in mecklenburg-Western Pomerania. Germany. *PLoS One* 10:e0143326. 10.1371/journal.pone.0143326 26606146 PMC4659621

[B7] Djordjevic S. P. Jarocki V. M. Seemann T. Cummins M. L. Watt A. E. Drigo B. et al. (2024). Genomic surveillance for antimicrobial resistance — a One Health perspective. *Nat. Rev. Genet*. 25, 142–157. 10.1038/s41576-023-00649-y 37749210

[B8] El-Halfawy O. M. Valvano M. A. (2015). Antimicrobial heteroresistance: An emerging field in need of clarity. *Clin. Microbiol. Rev*. 28, 191–207. 10.1128/CMR.00058-14 25567227 PMC4284305

[B9] Feldgarden M. Brover V. Gonzalez-Escalona N. Frye J. G. Haendiges J. Haft D. H. et al. (2021). AMRFinderPlus and the Reference Gene Catalog facilitate examination of the genomic links among antimicrobial resistance, stress response, and virulence. *Sci. Rep*. 11:12728. 10.1038/s41598-021-91456-0 34135355 PMC8208984

[B10] García-González N. Beamud B. Sevilla-Fortuny J. Sánchez-Hellín V. Vidal I. Rodríguez J. C. et al. (2025). Genomic surveillance reveals different transmission patterns between third-generation cephalosporin and carbapenem resistance in *Klebsiella pneumoniae* in the Comunidad Valenciana (Spain), 2018-2020. *Antimicrob. Resist. Infect. Control*. 14:44. 10.1186/s13756-025-01553-2 40336117 PMC12060429

[B11] Guido G. Frallonardo L. Asaduzzaman M. Farkas F. B. De Vita E. Seni A. et al. (2025). Third-generation Cephalosporin resistance in Sub-Saharan Africa: A systematic review and meta-analysis. *Commun. Med.* 6:10. 10.1038/s43856-025-01243-5 41258441 PMC12775075

[B12] Hafiz S. Oakley C. L. (1976). *Clostridium difficile*: Isolation and characteristics. *J. Med. Microbiol*. 9, 129–136. 10.1099/00222615-9-2-129 933146

[B13] Hendriksen R. S. Munk P. Njage P. van Bunnik B. McNally L. Lukjancenko O. et al. (2019). Global monitoring of antimicrobial resistance based on metagenomics analyses of urban sewage. *Nat. Commun*. 10:1124. 10.1038/s41467-019-08853-3 30850636 PMC6408512

[B14] Higgins P. G. Lehmann M. Wisplinghoff H. Seifert H. (2010). *gyrB* multiplex PCR to differentiate between *Acinetobacter calcoaceticus* and *Acinetobacter* genomic species 3. *J. Clin. Microbiol*. 48, 4592–4594. 10.1128/JCM.01765-10 20881170 PMC3008493

[B15] Hoffmann C. Zimmermann F. Biek R. Kuehl H. Nowak K. Mundry R. et al. (2017). Persistent anthrax as a major driver of wildlife mortality in a tropical rainforest. *Nature* 548, 82–86. 10.1038/nature23309 28770842

[B16] Jolley K. A. Bray J. E. Maiden M. C. J. (2018). Open-access bacterial population genomics: BIGSdb software, the PubMLST.org website and their applications. *Wellcome Open Res*. 3:124. 10.12688/wellcomeopenres.14826.1 30345391 PMC6192448

[B17] Lechleiter N. Wedemeyer J. Schütz A. Sehl-Ewert J. Schaufler K. Homeier-Bachmann T. (2025). Metagenomic analysis of the faecal microbiota and AMR in roe deer in Western Pomerania. *Sci. Rep*. 15:9288. 10.1038/s41598-025-93602-4 40102546 PMC11920406

[B18] Mahmud M. S. Hosen M. A. Hossion M. I. Sadik Sabuj M. S. Rumi N. A. Hossain M. K. et al. (2023). Isolation, identification, and characterization of resistant bacteria to antibiotics from pharmaceutical effluent and study of their antibiotic resistance. *Front. Microbiol*. 14:1307291. 10.3389/fmicb.2023.1307291 38230310 PMC10790235

[B19] Mammeri H. Sereme Y. Toumi E. Faury H. Skurnik D. (2025). Interplay between porin deficiency, fitness, and virulence in carbapenem-non-susceptible *Pseudomonas aeruginosa* and *Enterobacteriaceae*. *PLoS Pathog*. 21:e1012902. 10.1371/journal.ppat.1012902 39919103 PMC11805372

[B20] Murray C. J. L. E. A. (2022). Global burden of bacterial antimicrobial resistance in 2019: A systematic analysis. *Lancet* 399, 629–655. 10.1016/S0140-6736(21)02724-0 35065702 PMC8841637

[B21] Oberhofer T. R. (1979). Growth of nonfermentative bacteria at 42°C. *J. Clin. Microbiol*. 10, 800–804. 10.1128/jcm.10.6.800-804.1979 118175 PMC273274

[B22] Olsen N. S. Riber L. (2025). Metagenomics as a transformative tool for antibiotic resistance surveillance: Highlighting the impact of mobile genetic elements with a focus on the complex role of phages. *Antibiotics* 14:296. 10.3390/antibiotics14030296 40149106 PMC11939754

[B23] Prjibelski A. Antipov D. Meleshko D. Lapidus A. Korobeynikov A. (2020). Using SPAdes de novo assembler. *Curr. Protoc. Bioinformatics*. 70:e102. 10.1002/cpbi.102 32559359

[B24] Quince C. Walker A. W. Simpson J. T. Loman N. J. Segata N. (2017). Shotgun metagenomics, from sampling to analysis. *Nat. Biotechnol*. 35, 833–844. 10.1038/nbt.3935 28898207

[B25] Rempel O. R. Laupland K. B. (2009). Surveillance for antimicrobial resistant organisms: Potential sources and magnitude of bias. *Epidemiol. Infect*. 137, 1665–1673. 10.1017/S0950268809990100 19493372

[B26] Seliwiorstow T. De Zutter L. Houf K. Botteldoorn N. Baré J. Van Damme I. (2016). Comparative performance of isolation methods using Preston broth, Bolton broth and their modifications for the detection of *Campylobacter* spp. From naturally contaminated fresh and frozen raw poultry meat. *Int. J. Food Microbiol*. 234, 60–64. 10.1016/j.ijfoodmicro.2016.06.040 27391222

[B27] Tacconelli E. Carrara E. Savoldi A. Harbarth S. Mendelson M. Monnet D. L. et al. (2018). Discovery, research, and development of new antibiotics: The WHO priority list of antibiotic-resistant bacteria and tuberculosis. *Lancet Infect. Dis*. 18, 318–327. 10.1016/S1473-3099(17)30753-3 29276051

[B28] Vasimuddin M. Misra S. Li H. Aluru S. (2019). “Efficient Architecture-Aware Acceleration of BWA-MEM for Multicore Systems,” in *Proceedings of the 2019 IEEE International Parallel and Distributed Processing Symposium (IPDPS)*, (Rio de Janeiro), 314–324. 10.1109/IPDPS.2019.00041

[B29] Weller D. L. Murphy C. M. Love T. M. T. Danyluk M. D. Strawn L. K. (2024). Methodological differences between studies confound one-size-fits-all approaches to managing surface waterways for food and water safety. *Appl. Environ. Microbiol*. 90:e0183523. 10.1128/aem.01835-23 38214516 PMC10880618

[B30] WHO. (2017). *Global Antimicrobial Resistance Surveillance System (GLASS) Report: Early Implementation 2016-2017.* Geneva: World Health Organization.

[B31] WHO. (2021). *WHO Integrated Global Surveillance On ESBL-producing E. Coli Using a “One Health” Approach: Implementation and Opportunities.* Geneva: World Health Organization.

[B32] WHO. (2024). *WHO Bacterial Priority Pathogens List, 2024: Bacterial Pathogens Of Public Health Importance to Guide Research, Development And Strategies To Prevent And Control Antimicrobial Resistance.* Geneva: World Health Organization.

[B33] Wick R. R. Holt K. E. (2022). Polypolish: Short-read polishing of long-read bacterial genome assemblies. *PLoS Comput. Biol*. 18:e1009802. 10.1371/journal.pcbi.1009802 35073327 PMC8812927

[B34] Williams D. J. Grimont P. A. D. Cazares A. Grimont F. Ageron E. Pettigrew K. A. et al. (2022). The genus *Serratia* revisited by genomics. *Nat. Commun*. 13:5195. 10.1038/s41467-022-32929-2 36057639 PMC9440931

[B35] Wright G. D. (2010). Antibiotic resistance in the environment: A link to the clinic? *Curr. Opin. Microbiol*. 13, 589–594. 10.1016/j.mib.2010.08.005 20850375

[B36] Yamin D. Uskoković V. Wakil A. M. Goni M. D. Shamsuddin S. H. Mustafa F. H. et al. (2023). Current and future technologies for the detection of antibiotic-resistant bacteria. *Diagnostics* 13:3246. 10.3390/diagnostics13203246 37892067 PMC10606640

